# Selective cognitive dysfunction and physical disability improvement after autologous hematopoietic stem cell transplantation in highly active multiple sclerosis

**DOI:** 10.1038/s41598-020-78160-1

**Published:** 2020-12-04

**Authors:** N. Giedraitiene, R. Kizlaitiene, V. Peceliunas, L. Griskevicius, G. Kaubrys

**Affiliations:** 1 Clinic of Neurology and Neurosurgery, Institute of Clinical Medicine, Faculty of Medicine, Vilnius University, Vilnius, Lithuania; 2 Hematology, Oncology and Transfusion Medicine Center, Vilnius University Hospital Santaros Klinikos, Vilnius, Lithuania; 3 Faculty of Medicine, Institute of Clinical Medicine, Vilnius University, Vilnius, Lithuania

**Keywords:** Neurology, Neurological disorders, Demyelinating diseases, Multiple sclerosis

## Abstract

The aim was to assess the cognitive dysfunction and physical disability after autologous hematopoietic stem cell transplantation (AHSCT), to explore the potential factors influencing disability regression after AHSCT and to estimate the safety of low-dose immunosuppressive therapy in highly active Multiple Sclerosis (MS) patients. In single-center prospective study patients who failed to conventional therapies for highly active relapsing MS underwent the AHSCT. The disability was followed up with Expanded Disability Status Scale and cognition with Brief International Cognitive Assessment for Multiple Sclerosis. Twenty four patients [18 (72.0%) female] underwent AHSCT. Two patients of 13 had one relapse during the first year and three patients—during the second year after AHSCT. Disability regression was found in 84.6% of patients. The scores of information processing speed and verbal learning were significantly higher at month 12 after AHSCT. The clinical variable that explained the disability regression at months 6 and 12 after AHSCT was the disability progression over 6 months before AHSCT. No transplant related-deaths were observed. Selective cognitive improvement was found after AHSCT in MS patients. The disability may be temporarily reversible after AHSCT in a significant proportion of highly active RMS patients if AHSCT is well-timed performed.

## Introduction

Multiple Sclerosis (MS) is the most prevalent chronic autoimmune disease of the central nervous system (CNS) and is presumed to be the immune-mediated disease caused by autoreactive T cells^1^. Currently there are many disease-modifying treatments (DMT) for MS, and all of them are focused on the active inflammation control^2^. The evidence shows that the most effective disease-modifying therapy can help to prevent the relapses and to delay long-term disability progression in MS^2,3^. Despite advances in the current treatment of MS, some patients do not respond to available drugs and require other therapeutic strategies to control the disease activity and to prevent the progression of disability^2–4^.

During the last 25 years, severe autoimmune diseases including MS have been treated with immunosuppression and autologous hematopoietic stem cell transplantation (AHSCT)^5,6^. It enables to remove disease-causing immune cells and to reset the immune system^2^. Since 1995 more than 1000 patients with MS have undergone this treatment. Most of these patients were recognized as having highly active MS after failure of all available MS treatments^6–8^. Consensus recommendations on AHSCT as a second line therapeutic option for severe MS were published in 2012 and revised recently in 2019^9^. The recommendations recognized severe deteriorating MS patients despite standard therapy as the best candidates for AHSCT^9,10^. AHSCT ensures higher rates of disease activity (NEDA) than those achieved with any other DMT. AHSCT is also associated with greater short-term risks witch have limited its use in whom the disease can be controlled with DMT^11^.

The first clinical trials have addressed the issues of safety and efficacy of AHSCT with high-dose and intermediate-intensity [BEAM (conditioning regimen with carmustine, etoposide, cytarabine and melphalan)] conditioning regimen in MS resulting in clinical benefit^12–16^. Thereafter, it was shown that intermediate intensity, non-myeloablative regimen using cyclophosphamide with Anti-Thymocyte Globulin (ATG) is associated with similar efficacy but less toxicity. Less-intensive conditioning regimen with AHSCT is supported by some evidence that AHSCT is not only immunosuppressive therapy but also have an immunomodulatory component^9,17–19^.

The first AHSCTs were performed in patients with advanced progressive disease. Currently AHSCT is performed in patients with highly active disease course without adequate response to second line treatment, shorter disease duration and less disability^20–22^. The benefit in progressive forms of the disease is more limited, however, patients with higher degrees of disability, or occasionally progressive disease course, can be considered if there is clear evidence of significant clinical and MRI disease activity^19,23^.

The main concern limiting the use of AHSCT in MS patients is the mortality risk^21,24^. However, treatment-related mortality, which was initially high (3.6%), has decreased to 0.3% in studies post-2005. The reduction in treatment mortality appeared due to the greater experience, improvements in transplant techniques and optimization of patient selection^24^. The most common side effects of AHSCT are secondary to the immunosuppression and are tending to appear during the first 6 months after AHSCT. The most common complications include infections, febrile neutropenia, sepsis and viral reactivations. Late adverse events have also been described, including secondary autoimmune conditions, malignancies and infertility^25,26^.

For a long time, there was a lack of randomized clinical trials comparing AHSCT with DMT in MS patients^19,22,27,28^. Recently the first randomized clinical trial has shown the higher efficacy of AHSCT versus DMT in relapsing–remitting MS. In this study AHSCT compared with DMT resulted in prolonged time to disease progression^27^. However, further research is needed to replicate these findings^27^ and to assess long-term outcomes and safety of AHSCT. Experience from single centers and long-term follow-up of MS patients remain important in providing valuable information about the efficacy and safety of AHSCT. In this single-center prospective observational cohort study we describe disease characteristics and outcomes of the Lithuanian population treated with AHSCT, between 2014 and 2019 years.

The main objective of our study was to assess the cognitive dysfunction and physical disability after AHSCT, to explore the potential factors influencing disability regression after AHSCT and to estimate the safety of low-dose immunosuppressive therapy (LDIT) in highly active MS patients.

## Results

### Patients’

Twenty-four patients were selected and underwent AHSCT procedure. All patients had relapsing–remitting MS. The mean follow-up was 22.3 ± 16.5 months (range 5–77). Demographic and clinical characteristics of the patients are provided in Table 1.

**Table 1:** Numerical and percentage distribution of patients with MS undergoing AHSCT, according to demographic and clinical characteristics.

Demographic and clinical variables	N	%
**Sex**		
Female	18	72
Age (years)	37.8 ± 5.9	–
Disease duration (years)	8.6 ± 5.5	–
Education (years)	16 ± 3.7	–
**EDSS**		
12 months before AHSCT	4.4 ± 1.1	–
6 months before AHSCT	4.8 ± 0.9	–
Before AHSCT	5.9 ± 0.8	–
**Previous DMT**^**a**^		
Fingolimod	9	37.5%
Natalizumab	12	50.0%
Alemtuzumab	1	4.2%
Interferon -beta	2	8.3%
**Number of previously used DMT**		
2	11	28.2%
3	8	20.5%
4	3	7.7%
5	2	5.1%

*AHSCT* autologous hematopoietic stem cell transplantation, *EDSS* expanded disability status scale, *DMT* disease modifying treatment.

^a^The last medication is provided.

**Figure 1:**
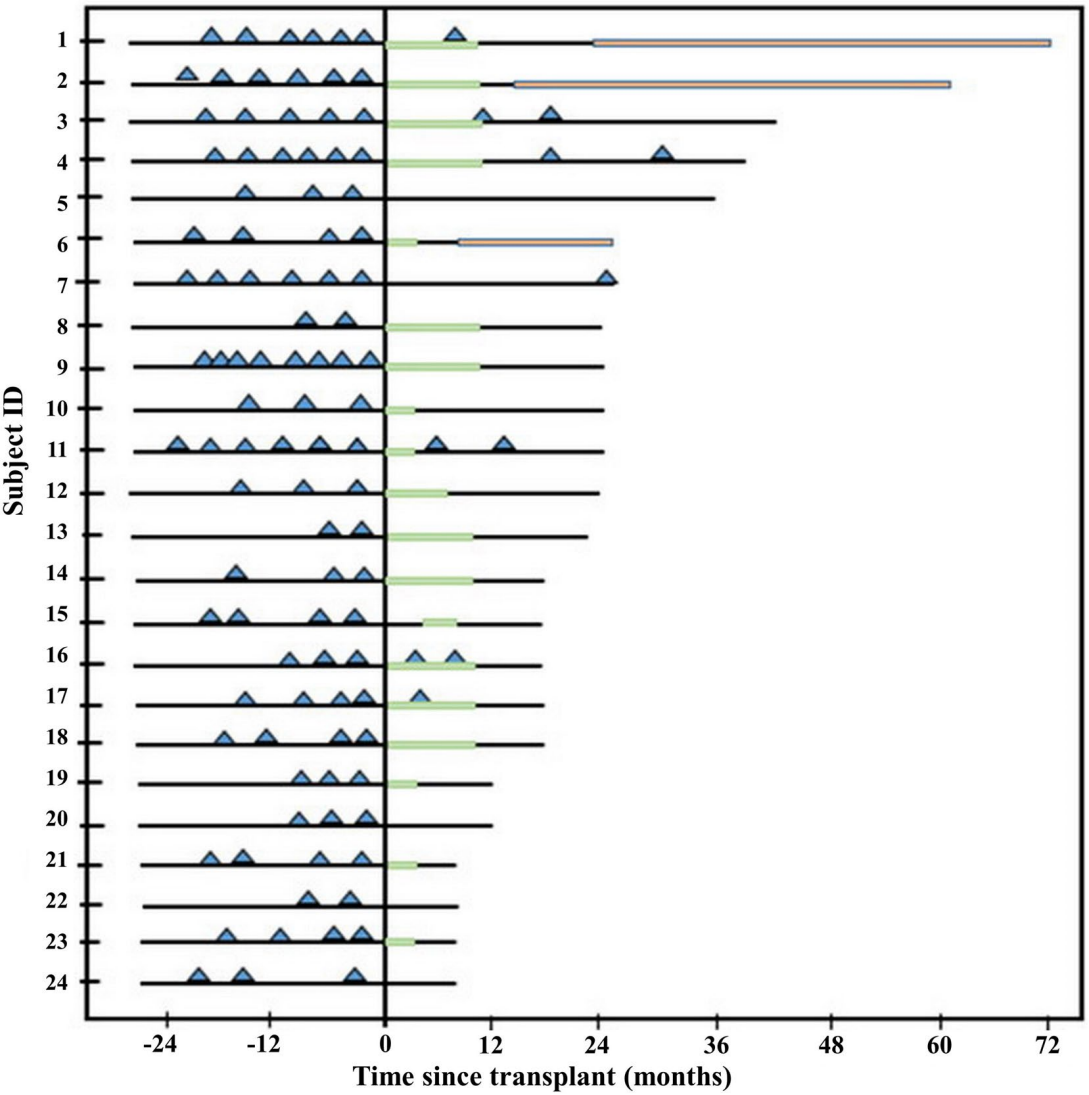
Patient level pretransplant disease characteristics and outcomes.  MS relapse,  disability progression,  disability regression.

Patient pretransplant disease characteristics and outcomes after AHSCT are shown in Fig. 1.

13 patients (54.2%) of 24 patients who underwent AHSCT completed the 24 month follow-up and were included in the efficacy analysis. As potential side effects can occur during the 6 months after AHSCT^23,25,26^, the safety issues were analysed in all 24 patients who completed 6 month follow-up.

### Efficacy and outcome

#### Relapses and annualized relapse rate

**Figure 2:**
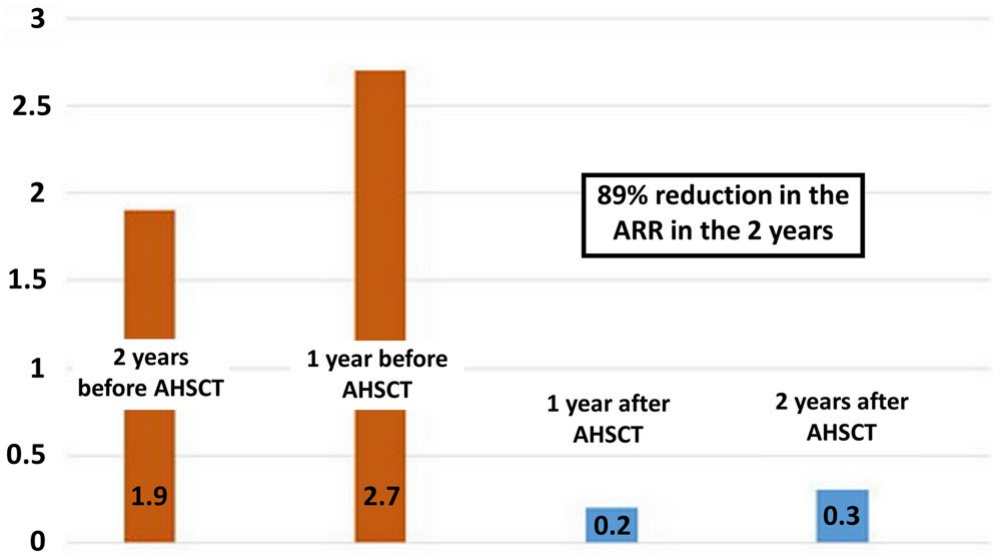
Annualized relapse rate in 2 years before AHSCT and in 2 years after AHSCT. *AHSCT* autologous hematopoietic stem cell transplantation, *ARR* annualized relapse rate. Data on relapses and ARR were assessed in patients who completed 24 month follow-up (N = 13).

Two patients (15.4%) of 13 had one relapse during the first year after AHSCT and three patients (23.1%) had one relapse during the second year after AHSCT. The annualized relapse rate (ARR) dropped to 0.2 in the first year and to 0.3 in the second year. A reduction of 89% was found when comparing the ARR 2 years after AHSCT (0.5) with ARR 2 years before AHSCT (4.6) (Fig. 2).

#### Disability progression and improvement

**Figure 3:**
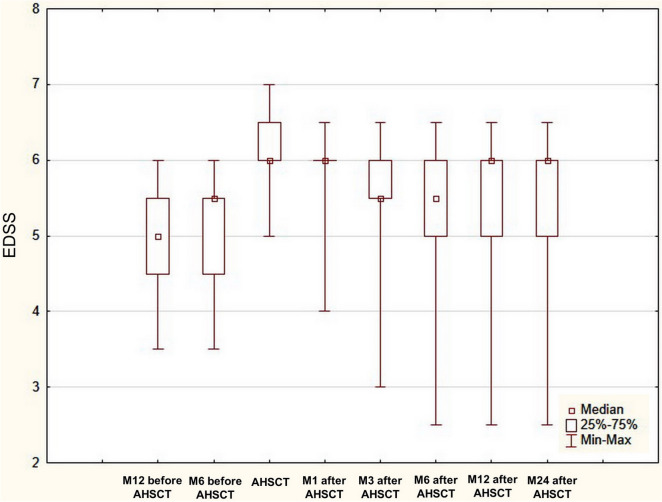
Changes in EDSS score from one year before to two years after AHSCT. *EDSS* expanded disability status scale, *AHSCT* autologous hematopoietic stem cell transplantation, *M1* month 1, *M3* month 3, *M6* month 6, *M12* month 12, *M24* month 24. Disability change was assessed in patient who completed 24 month follow-up (N = 13).

An improvement in EDSS score at least by 0.5 points at month 6 from baseline was observed in 11 patients (84.6%) after AHSCT: in six patients (46.2%) the EDSS score improved by 0.5 (EDSS score in these patients was > 5.5), in two patients (15.4%)—by 1.5, in two patients (15.4%)—by 2.0 and in 1 patient (7.8%)—by 2.5 points. Sustained disability improvement was found in 10 (76.9%) of 13 patients at month 12 after AHSCT. In 10 patients (76.9%) of 13 EDSS score remained stable at month 24 after AHSCT, in one patient (7.7%) improved by 0.5 and in two patients (15.4%) by 1.0 point (Fig. 3).

#### Cognition after AHSCT

The score of Symbol Digit Modalities Test (SDMT) that assesses information processing speed was slightly lower at month 3 than before AHSCT, however, the difference was not significant. The score of SDMT was significantly higher at month 12 than before AHSCT and at month 3. And the score of SDMT was significantly lower at month 24 than at month 12. The score of Brief Visuospatial Memory Test Revised (BVMT-R) that assesses visuospatial memory was slightly lower at month 3 than before AHSCT, however, the difference was not significant. The scores of BVMT-R didn’t differ between pretransplant, month 12, and month 24 after AHSCT assessments. The score of California Verbal Learning Test Second Edition (CVLT-II) that assesses verbal learning was significantly higher at month 12 than before AHSCT, while the scores of CVLT-II didn’t differ between pretransplant assessment and month 3, also between month 12 and 24 after AHSCT (Table 2).

**Table 2:** Cognitive scores from before AHSCT to 2 years after AHSCT.

Test	M0	M3	M12	M24	rmANOVA	Post-hoc
SDMT	51.4 ± 3.8	48.3 ± 3.5	56.8 ± 2.7	49.1 ± 4.1	F = 10.9 *p* < 0.001	M12 > M0, M3, M24 M0 = M3 = M24
BVMT-R	27.8 ± 4.9	25.6 ± 2.8	28.1 ± 5.6	26.9 ± 5.1	F = 3.2 *p* > 0.05^a^	M0 = M3 = M12 = M24
CVLT-II	55.2 ± 5.9	56.0 ± 6.6	63.6 ± 8.4	59.2 ± 11.6	F = 5.6 *p* < 0.01^a^	M0 = M3 < M12 = M24

*rmANOVA* repeated measures analysis of variance, *SDMT* symbol digit modalities test, *BVMT-R* brief visuospatial memory test revised, *CVLT-II* California verbal learning test second edition, *AHSCT* autologous hematopoietic stem cell transplantation, *M0* before AHSCT, *M3* month 3, *M12* month 12, *M24* month 24.

Cognitive scores were assessed in patient who completed 24 month follow-up (N = 13).

^a^Greenhouse–Geisser criterion was used to correct the violation of sphericity.

#### MRI assessment

Eight patients (61.5%) of 13 had new or enlarged T2 lesions and eight patients (61.5%) had active lesions on the brain MRI before AHSCT. No new or active lesions were found on the spinal MRI before AHSCT. There were no new or enlarged T2 lesions, no gadolinium-enhanced lesions on the brain and spinal MRI in 13 patients that have completed 24 month follow-up at month 3, 12, 24 after AHSCT.

#### Analysis of variables that explained the disability regression after AHSCT

General Linear Model (GLM) was used to assess the impact of demographic characteristics and disease characteristics on the disability change after AHSCT per 6 months and 12 months. Dependent variables in the models were—EDSS change from baseline assessment before AHSCT to 6 months after AHSCT (∆EDSS_B-6_) or EDSS change from baseline assessment before AHSCT to 12 months after AHSCT (∆EDSS_B-12_). Independent variables (regressors) in the models were—age, gender, disease duration, change in EDSS score from 6th month before transplant to baseline assessment (∆EDSS_6-B_) or change in EDSS score from 12th month before transplant to baseline assessment (∆EDSS_12-B_) (one of them per model), relapse number per 6 months before transplant (RN6) or relapse number per 12 months before transplant (RN12), either relapse number per 24 months before transplant (RN24) (one of them per model), the score of one of the Brief International Cognitive Assessment for Multiple Sclerosis (BICAMS) tests (SDMT or BVMT, either CVLT-II), the number of new and active lesions on brain MRI.

Regression models that had explained the disability improvement after AHSCT:

1. ∆EDSS_B-6_ = −1.5 + 0.79x (∆EDSS_6-B_); R^2^ = 0.55; *p* < 0.05.
2. ∆EDSS_B-12_ = −1.2 + 0.54x (∆EDSS_6-B_); R^2^ = 0.48; *p* < 0.05.

The model was considered successful if both the model itself and all the independent variables left in the model by the backward removal procedure were significant (*p* < 0.05).

### Side effects of AHSCT procedure

Conditioning regimen induced myelosupression with profound pancytopenia in all patients. All patients received platelet transfusions, majority red blood cells transfusions. Febrile neutropenia (FN) was diagnosed in 19 patients (79%) and was most common adverse event after AHSCT. 10 FN episodes developed within 48 h of stem cell reinfusion and were probably a reaction to circulating ATG. Blood culture was positive only in one FN episode. One patient developed Acute respiratory distress syndrome (ARDS) with respiratory failure during conditioning. ARDS resolved after treatment with glucocorticoids. During the follow-up period one patient developed idiopathic thrombocytopenic purpura (ITP), not responsive to glucocorticoids and normal human immunoglobulin therapy. The previous DMT in this patient was fingolimod. Third line therapy with eltrombopag in this patient resulted in prompt response. Eltrombopag was discontinued after 3 months, platelet count remained within normal range. One patient developed hemorrhagic cystitis of viral etiology [significant Adenovirus (ADV) and John Cunningham virus (JCV) viruria detected]. Cystitis resolved after infusion of normal human immunoglobulin. Epstein–Barr virus (EBV) reactivation was detected in 18 (75%) patients during follow-up period. Median number of EBV copies/ml was 1145, range 32–223,000. 5 patients were considered at risk of postransplant lymphoproliferative disorder (PTLD) and received preemptive treatment with rituximab. EBV reactivation resolved in all patients. There were no PTLD cases in our cohort. All adverse events associated with AHSCT procedure resolved. All patients are alive.

## Discussion

The previous studies have shown that patient selection is important in determining outcomes of AHSCT for MS patients and highly active relapsing MS is the target population for recent investigations^6,13,15,16^. Among the patients involved in the earlier studies, the patients with relapsing–remitting forms of MS demonstrated more favourable results than those with progressive MS forms (both primary progressive and secondary progressive) without activity^7,8,12^. The significant difference is possibly due to the predominant neuroinflammatory process in relapsing forms of MS^1,4^. On the basis of the previous studies we included only the highly active relapsing MS patients with clinical evidence of disease activity without the response to currently available MS therapy. Most patients (91.7%) failed on the second line MS therapy, therefore the study represented highly active RMS patients.

Since the first transplantations in MS were performed and the data published, several protocols for mobilization and conditioning have been used^6–8,10^. Intermediate intensity, non-myeloablative conditioning regimen at our center was selected over high intensity regimens due to superior safety and comparable efficacy^12–16^. The main reason for using intermediate intensity regimen instead of high intensity regimen was to avoid transplant related mortality.

The primary goal of AHSCT is to suppress the active disease and to prevent further disability progression^5,9,10^. The main result in the terms of efficacy in our study was the dramatic reduction in the relapses and annualized relapse rate in the 2 years after AHSCT and the reduction of disability progression, with 84.6% of patients improving their disability score after AHSCT at month 6 and 76.9%—at month 12. Since most patients who progressed before AHSCT subsequently improved in EDSS disability at month 6 and 12, the current definitions of disability progression for relapsing–remitting MS may not accurately estimate irreversible disease progression for all patients^29^. No new or active lesions were found on MRI after AHSCT, this meant that all patients remained without radiological disease activity. These outcomes are highly promising, as compared to conventional MS treatment outcomes^2,4^, and consistent with other investigations of AHSCT for similar MS population^13,14,20–22^.

Current available MS treatment may delay or prevent further increase in disability^2–4^. Few studies have published the data concerning the ability of MS therapies to help to reduce the disability at least temporarily^30–32^, however, there is a need for suitable analytical methods to assess this novel outcome. So far there is a lack of randomized clinical trials comparing the efficacy of AHSCT with DMT in MS patients^24,28^. Recently the first randomized clinical trial have demonstrated the higher efficacy of AHSCT versus DMT in relapsing–remitting MS^27^. However, further research is needed to replicate these findings and to assess long-term outcomes and safety of AHSCT^27^. Mainly the evidence of effectiveness from the large registry studies indicates that AHSCT is of the highest effectiveness in highly active RMS^8,13,22^. The current analysis shows that in patients with RMS with inadequate response to prior DMT, AHSCT provides not only a dramatic reduction in relapse rate, but also temporary disability regression. The findings of EDSS improvement in almost 85% of the patients suggest that disability may be often at least temporarily reversible in patients with highly active RMS if they receive suitable and well-timed treatment irrespective of the severity of baseline deficit. On the other hand, the effect of the AHSCT on the disability regression after transplantation can be explained by the severity of disability progression before the transplantation and well-timed transplantation can be effective in highly active MS patients to the extent that it causes disability regression.

The EDSS is most commonly used and recognized neurological disability assessment tool in the clinical trials, however, it doesn’t adequately reflect cognition^33^. The BICAMS that reliably assesses three cognitive domains^39,40^ was selected to provide more elaborate cognitive evaluation. Non-significant decline in information processing speed and visuospatial memory was found at month 3 after AHSCT and significant improvement of information processing speed at month 12 after AHSCT. The results at month 3 were not significant, however, slight decline in information processing speed and visuospatial memory can suggest that AHSCT using intermediate intensity lymphoablative conditioning regimen may negatively impact the cognitive function most likely information processing speed and visuospatial memory in the early phase after the AHSCT procedure. Our results supports the previously published data^34,35^ that AHSCT procedure have the temporal negative impact on the cognition, however, despite it’s aggressive nature do not have lasting deleterious effect on it. The improvement in information processing speed and verbal learning at month 12 in our study confirms that selective cognitive improvement can occur after AHSCT in MS patients.

The incidence and severity of adverse events after AHSCT were in the expected range and similar to the data reported in the literature^6–8,10,12^. There were no transplant-related deaths. Our study data add to the already considerable body of evidence supporting the usefulness of AHSCT and its favorable benefit-to-risk profile in the treatment of highly active RMS when all available DMTs have failed.

The main limitation of the study was the relatively low sample size, therefore, the results of the study cannot be generalized and further research is needed to replicate these findings. On the other hand, the patient’s assessment and follow-ups were provided at the same-center without comparative group. However, we did not identify any controlled study, with the exception of one small randomized controlled trial^27^ in which comparative group is used to assess the results of AHSCT^7,8,12,22^. The absence of comparative group remains the main limitation in most AHSCT studies in MS. Therefore, the experiences from single centers and sustained long-term follow-up of MS patients remain important in providing valuable information about the efficacy and safety of AHSCT.

## Methods

The Lithuanian Bioethics Committee approved the study in 2011 (2011-01-27 No.: L-12-01/2), the permission to continue the study was granted by the Lithuanian Bioethics Committee in 2018 (2018-02-22 No.: 6B-18-41). All methods were performed in accordance with the relevant guidelines and regulations. The open-label prospective single-center observational cohort study was conducted in Vilnius University Hospital Santaros Klinikos, Lithuania. The patients were enrolled in the study between 2013 and 2019. All participants signed the Informed Consent Form. The AHSCT procedure is performed at our hospital as routine clinical practice for highly active relapsing MS patients who do not respond to second line therapy. Highly active MS patients in the case of AHSCT were defined the patients who experienced at least two relapses and had disability progression of at least 2.0 points according EDSS in the last year. Fingolimod, cladribine, natalizumab, ocrelizumab and alemtuzumab are the second line therapy for MS in Lithuania based on the regulations of the Ministry of Health. Exclusion criteria were primary or secondary progressive MS; neurological disorders, other than MS; active infections; pregnancy; pulmonary, cardiac, renal, or liver dysfunction; abnormal blood cell counts. The AHSCT procedure was carried out in Hematology, Oncology and Transfusion Medicine Center of Vilnius University Hospital Santaros Klinikos, Vilnius, Lithuania.

Participants underwent peripheral blood stem cells mobilization: cyclophosphamide (2 g/m^2^ single dose with intravenous mesna prophylaxis) was administered, subcutaneous filgrastim 10 µg/kg was started on day +7 and peripheral blood stem cell (PBSC) apheresis procedure were targeted on day +12 after cyclophosphamide. Collected cells were cryopreserved using dimethyl sulfoxide (DMSO) and stored in liquid nitrogen. The target yield of cryopreserved CD34+ cells was > 2.5 × 106 CD34+/kg. The conditioning regimen was intravenous cyclophosphamide, 50 mg/kg per day on days − 5 to day − 2 (total dose 200 mg/kg) and rabbit antithymocyte globulin 0.5 mg/kg on day − 5, 1.5 mg/kg on day − 4, and 1.5 mg/kg on days − 4, − 3, − 2, and − 1 (total dose 6.5 mg/kg). Methylprednisolone (1000 mg) was infused 30 min prior to rabbit antithymocyte globulin infusion. Hydration (125–150 mL normal saline per hour), diuretics, and intravenous mesna were continued until 24 h after the last dose of cyclophosphamide. Filgrastim (5–10 µg/kg per day) was started on day +5 and continued until engraftment. Cytomegalovirus (CMV) and Epstein–Barr virus (EBV) was monitored by PCR for at least 90 days after AHSCT.

All MS patients were diagnosed with Multiple Sclerosis according to McDonald criteria by a neurologist of the Vilnius Multiple Sclerosis Center^36^. MS relapses were determined by the examining neurologist and were diagnosed when neurologic symptoms lasted more than 24 h and occurred at least 30 days after the onset of a preceding relapse and were not associated with any other trigger^37^. Information about relapses per 6 months, 1 and 2 years before AHSCT were collected from the National Multiple Sclerosis Registry. ARR was defined as the total number of relapses divided by the total person-time at risk of relapse.

Neurological disability was assessed with EDSS^38^. The score on the EDSS was recorded prior the AHCST, at 1 month and every three months after AHSCT. Data from EDSS score 6 months, 1 and 2 years before AHSCT were obtained from the National Multiple Sclerosis Registry. Confirmed disability progression was defined as an increase in EDSS score of at least one point from baseline EDSS was ≤ 5.5 points, or an increase of ≥ 0.5 points if the baseline EDSS was > 5.5 points and confirmed disability regression as a decrease of at least one point from baseline EDSS was ≤ 5.5 points, or an increase of ≥ 0.5 points if the baseline EDSS was > 5.5 points.

Brain and spinal cord magnetic resonance imaging (MRI) with Gadolinium were performed before AHSCT, at 3 month and every year after AHSCT. MRI in all patients was performed using a 3.0 T scanner Philips ACHIEVA 3TX. MRI assessment included the following sequences: T1 (repetition time 526 ms, echo time 14 ms), T2 (repetition time 4110 ms, echo time 105 ms) and fluid-attenuated inversion recovery (FLAIR) T2 (repetition time 9000 ms, echo time 122 ms). MRI was assessed by one and the same radiologist.

Cognition was assessed with Brief International Cognitive Assessment for Multiple Sclerosis (BICAMS)^39–42^. All subjects were assessed by the same person, in the same assessment room and in the same BICAMS test sequence: the Symbol Digit Modalities Test (SDMT), the Brief Visuospatial Memory Test Revised (BVMT-R), the California Verbal Learning Test, second edition. Parallel versions of SDMT, BVMT-R and CVLT-II were used in each evaluation session^42^. The cognitive assessment was done before AHSCT, at 3 month and every year after AHSCT.

Patients were followed by the transplant physician for at least 6 months after AHSCT. CMV and EBV PCR tests were performed routinely for at least 6 months after AHSCT. We collected information about all nonhematological adverse events grade ≥ II° from the start of mobilization to 6 months after AHSCT.

### Statistical analysis

Data were analyzed using statistical software package SPSS (version 20.0 for Windows). Continuous variables were reported as medians and ranges or means and standard deviations, while categorical variables were reported as absolute numbers and percent of total patients. GLM Repeated Measures were used when measuring the data of cognitive scores at different time points (before AHSCT, at month 3, month 12 and month 24). General linear regression was used to assess the impact of various demographic, clinical factors on the disability change after AHSCT per 6 months and 12 months. A significance level *p* < 0.05 was accepted.
